# Assessing the Quality of Heart Rate Variability Estimated from Wrist and Finger PPG: A Novel Approach Based on Cross-Mapping Method

**DOI:** 10.3390/s20113156

**Published:** 2020-06-02

**Authors:** Mimma Nardelli, Nicola Vanello, Guenda Galperti, Alberto Greco, Enzo Pasquale Scilingo

**Affiliations:** 1 Bioengineering and Robotics Research Centre E. Piaggio, University of Pisa, 56122 Pisa, Italy; m.nardelli@ing.unipi.it (M.N.); nicola.vanello@unipi.it (N.V.); alberto.greco@unipi.it (A.G.); 2 Dipartimento di Ingegneria dell’Informazione, University of Pisa, 56122 Pisa, Italy; guendagalperti@gmail.com

**Keywords:** photoplethysmographic (PPG) signal, pulse rate variability (PRV), heart rate variability (HRV), cross-mapping, kurtosis, Shannon entropy, signal quality indexes, phase space reconstruction, wearable monitoring systems

## Abstract

The non-invasiveness of photoplethysmographic (PPG) acquisition systems, together with their cost-effectiveness and easiness of connection with IoT technologies, is opening up to the possibility of their widespread use. For this reason, the study of the reliability of PPG and pulse rate variability (PRV) signal quality has become of great scientific, technological, and commercial interest. In this field, sensor location has been demonstrated to play a crucial role. The goal of this study was to investigate PPG and PRV signal quality acquired from two body locations: finger and wrist. We simultaneously acquired the PPG and electrocardiographic (ECG) signals from sixteen healthy subjects (aged 28.5 ± 3.5, seven females) who followed an experimental protocol of affective stimulation through visual stimuli. Statistical tests demonstrated that PPG signals acquired from the wrist and the finger presented different signal quality indexes (kurtosis and Shannon entropy), with higher values for the wrist-PPG. Then we propose to apply the cross-mapping (CM) approach as a new method to quantify the PRV signal quality. We found that the performance achieved using the two sites was significantly different in all the experimental sessions (*p* < 0.01), and the PRV dynamics acquired from the finger were the most similar to heart rate variability (HRV) dynamics.

## 1. Introduction

Heart rate variability (HRV) is a reflection of the extrinsic regulation of heart rhythm and represents a robust noninvasive tool for observing the interplay between the two main branches of the autonomic nervous system (ANS), i.e., the sympathetic and parasympathetic nervous systems. Since the heart rate is actually a nonstationary phenomenon, HRV expresses the variation over the time of the period between two successive heartbeats. By studying HRV signals, it is possible to collect prognostic information to characterize the status of ANS, but also diagnostic information by detecting the early onset of cardiovascular diseases, e.g., congestive heart failure and myocardial infarction [1,2]. Previous literature reported that mood and emotional changes can also influence ANS dynamics, and HRV has been considered a promising marker of general psycho-behavioral response to internal and external stimuli [3,4,5]. The gold standard procedure used to extract the HRV consists of measuring the time intervals between each pair of consecutive R peaks from an electrocardiographic (ECG) signal. The period between two successive R-peaks temporally includes a cardiac cycle. The R peaks are particularly suitable for automated detection by computer algorithms, given their distinct profile with respect to the rest of the ECG signal.

In the last decades, photoplethysmography (PPG) technique is finding widespread use, leveraging upon the progress in optoelectronics and semiconductor technologies. Specifically, PPG sensors can be easily integrated into wearable devices, which are less obtrusive than ECG acquisition systems and can be used for clinical purposes, fitness monitoring, and research experiments [6,7,8]. PPG signal describes the changes in the light intensity emitted by a photo-emitter, absorbed or reflected when traveling through biological tissues and bloodstream. These variations reflect changes in blood volume which are coupled to heart electrical activity so that PPG waveform reflects the systole and diastole of the cardiac cycle. For this reason, HRV can also be extracted by measuring the time interval between two consecutive systolic pulses (pulse rate variability, i.e., PRV) [9]. According to the literature, features extracted from PRV signals constitute a promising tool for the early diagnosis and quantification of cardiovascular issues. Being the PPG signal is a good candidate for long-term acquisitions with wearable and portable systems, novel processing approaches can have a huge utility in early identification of hypovolemia in out-of-hospital settings [10,11]. Furthermore, PPG/PRV monitoring can serve as a tool for management of concussion associated to cardiovascular symptoms (for example in athletes during contact sports) [12].

However, on the one hand technology has successfully managed to increase the comfort, minimizing the weight and the size and maximizing the battery life of wearable sensors, on the other hand scientific research is working on the issue regarding corruption of recorded signals mainly due to motion and noise artifacts and poor sensor adhesion [13,14]. Considering the great versatility of PPG technology, one of the main topics of recent research in signal processing is to maximize its potential, identifying when the quality of a PPG signal is high enough to be used to derive an acceptable substitute of HRV.

The study of PPG signal quality can be performed at two different levels: (i) quantifying the quality of the PPG signal itself, (ii) measuring the reliability of PRV derived from PPG as a substitute for HRV extracted from ECG. In both analyses the PPG sensor location plays a crucial role, because different body sites are characterized by distinct tissue thickness, vascularization, and skin pigmentation which influence the shape of the waveform [15]. Fingers, earlobes, forehead, and wrist are common PPG recording locations. Finger is the body site most used for clinical applications, being the most practical and the most sensitive to blood volume fluctuations [16,17,18]. From quantitative analysis of waveform characteristics, e.g., mean amplitude and peak point position, the forehead resulted to be related to the least analyzable PPG signal, whereas the finger and the earlobe were reported to be the locations which produce the most reliable PPG signals [17]. The same results were found using statistical approaches, e.g., kurtosis and Shannon entropy, to automatically quantify the quality of multi-site PPG signals [17,19,20].

Concerning the investigation of the similarity of the PRV and HRV signals acquired at the same time, most of the studies in the literature compared the RR and PP interval series directly, using measures of statistical correlation [21,22]. Another common strategy was to compare the values of standardized features extracted from HRV and PRV signals in time and frequency domains [1,21,22,23,24]. However, the time spent on choosing the features, computing them and analyzing the results was high, the findings were controversial and task-dependent [21,25,26]. These disadvantages were especially due to the absence of a unique index which could quantify the PRV signal quality.

In this study, we compare PPG signals acquired from two wearable and portable devices, i.e., one finger-worn device (Shimmer 3 GSR+ [27]) and one wrist-worn device (Empatica E4 [28]), during an experimental protocol of emotion elicitation through visual stimuli. We chose to compare the gold-standard location for PPG acquisition, i.e., the finger, with the wrist considering the practical advantages of using novel wearable commercial devices, e.g., smartwatches. Wrist-worn PPG devices are more comfortable and less invasive than finger-worn acquisition systems, allowing long-term monitoring during daily activity. Moreover, the wrist is a location not affected by the body auto-regulation mechanisms, which can represent a confounding factor when finger location is used to acquire PPG. PPG signal quality was compared computing two well-known statistical indexes: kurtosis and Shannon entropy [17,19,20]. Then, we compare the two PRV signals with the HRV acquired during the experiment by using a wearable ECG acquisition system from Smartex s.r.l. [29]. For this purpose, we propose a novel approach based on Takens’ phase space reconstruction theory [30] and cross-mapping methodology [31,32]. The processes in the upper layers of the tissues involved in the PPG acquisition have been demonstrated to create chaos, therefore PPG dynamics, as well as ECG dynamics, can be considered chaotic [33]. Applying cross-mapping approach, we start from the hypothesis that the attractors traced by PRV and HRV points in the phase space describe the same complex dynamics of the cardiovascular system, and we suggest to trace the HRV attractor using the information collected from the PRV trajectories. The level of agreement between the real HRV and the surrogate series derived from the emulated attractor uniquely indicates the PRV signal quality. Finally, statistical analysis is carried out in order to investigate differences between the quality of PPG and PRV signals acquired from the two body sites and across the experimental sessions.

## 2. Materials and Methods

### 2.1. Experimental Protocol

Sixteen healthy subjects (aged 28.5 ± 3.5, seven females) performed an experimental protocol of passive emotion elicitation through visual stimuli. Each participant gave his/her informed consent to take part in the study, self-reporting no history of clinical and sub-clinical diseases, and absence of mental and personality disorders. During the whole duration of the experiment, participants were comfortably seated and wore earplugs in order to prevent any auditory cues. This study was approved by the Ethical Committee of the University of Pisa.

The affective elicitation was performed by visualizing pictures selected from the International Affective Picture System (IAPS) database [34] onto a PC monitor. Each picture belonging to IAPS database is characterized by two scores according to the Circumplex Model of Affect [35], with respect to different combinations of valence (pleasantness/unpleasantness) and arousal (intensity of the elicited emotion). The slide-show included four sessions lasting one minute, and each session consisted in ten images of 6 seconds. The order of the experimental sessions was the following:

n1: A first session of neutral images;
N: A session of images with high arousal and negative valence;
P: A session of images with high arousal and positive valence;
n2: A second session of neutral images.

During the experimental protocol, ECG signals of the participants were acquired by means of a textile-based sensorized t-shirt embedded with electrodes developed by Smartex s.r.l., with a sampling rate of 250 Hz. From each participant, two PPG signals were acquired from two different sites: a wrist-PPG signal ($PPG_{w}$) recorded through Empatica’s E4 wristband (sampling rate of 64 Hz) from the left wrist, and a finger-PPG signal ($PPG_{f}$) monitored using the Shimmer 3 GSR+ (sampling rate of 128 Hz) from the left index finger. During the following processing steps of PPG signals, that from finger was downsampled to 64 Hz in order to have the same sampling rate for both $PPG_{f}$ and $PPG_{w}$ signals. PPG signals were filtered using a second-order 0.3–10 Hz Butterworth band pass filter [36,37]. Ectopic beats were corrected through the use of Kubios HRV software [38]. A shape-preserving piecewise cubic interpolation at the standard rate of 4 Hz was applied to all interbeat (RR) and interpulse (PP) series.

### 2.2. PPG Signal Quality Quantifiers

As signal quality indexes, we estimated kurtosis and the Shannon entropy. Kurtosis is a measure related to the tailedness of data distribution. It is often used to evaluate the difference of the distribution tails with respect to the normal distribution. Specifically, if kurtosis is higher than 3 the data distribution is more peaked around the mean, while when it is lower it depicts a data distribution that is flatter than the normal one. Noticeably, kurtosis was found to increase in case of movement artifacts in PPG data [19] and it was proposed as a measure to design automatic approaches for PPG quality assessment [39].

Kurtosis can be measured as

(1) $$kurt=\frac{\sum_{i=1}^{N}{\left(x\left(i\right)-\mu_{x}\right)}^{4}}{{\left(\sum_{i=1}^{N}{\left(x\left(i\right)-\mu_{x}\right)}^{2}\right)}^{2}}$$

Shannon entropy is related to the amount of information contained in a signal. Its lower bound is related to the code length needed to describe the signal changes. For this reason, it was suggested as a measure to detect possible changes in the PPG related both to shape [20] or to artifact movements [19]. It is obtained by dividing the signal amplitude range in *k* bins. Given p(i), the estimate of probability for the signal to assume values within the $i^{th}$ bin, Shannon entropy can be measured as

(2) $$shannon=\sum_{i=1}^{n_{bins}}-p(i)log(p(i))$$

Both measures were estimated for each session 60 s long.

### 2.3. PRV Signal Quality Quantifiers

#### 2.3.1. Statistical Correlation

Considering each experimental session (n1, N, P, n2), statistical correlation between RR extracted from ECG and PP series was investigated computing the Pearson’s Linear Correlation Coefficient, specifically $r_{f}$ when RR is compared with $PP_{f}$ extracted from finger-PPG and $r_{w}$ when RR is compared with $PP_{w}$ extracted from wrist-PPG.

#### 2.3.2. Cross-Mapping

Cross-mapping method relies on Takens’ theory on the phase space reconstruction [30]. According to Takens’ theorem, a dynamical system can be described by its trajectories in the phase space, and the trajectory attractor can be reconstructed from one of the phase space coordinates alone, using time-delayed embedding. The embedding process depends on two parameters: the embedding dimension *E*, i.e., the dimension of the reconstructed phase space, and the time delay τ, used to find the coordinates of the points in the phase space. Starting from a time series *f* of length *n*, the n−(E−1)τ phase space vectors are defined as follows:

(3) F(t)=[f(t),f(t−τ),f(t−2τ),…,f(t−(E−1)τ)]

Let [x(1),x(2),…,x(n)] and [y(1),y(2),…,y(n)] be two time series representing the observation functions of the same dynamic process and describing diffeomorphic attractors X=X(1),X(2),…,X(L) and Y=Y(1),Y(2),…,Y(L), with L=n−(E−1). Sugihara et al. suggested that cross-mapping method can be used to generate an estimate of *Y* from the reconstructed manifold or “shadow manifold” $M_{X}$, derived from *X* [31,32]. The estimate of one point in the phase space of Y computed starting from the shadow manifold Mx, is called $\hat{Y}\left(t\right)|M_{X}$, and is performed through a simplex projection: a nearest-neighbor algorithm which uses exponentially weighted distances from nearby points on $M_{X}$ to compute a kernel density estimation of Y(t). Specifically, in order to find the cross-mapped estimate of the point $\hat{Y}\left(t\right)|M_{X}$ starting from the shadow manifold $M_{X}$, the corresponding point over time X(t) has to be identified in $M_{X}$, and a small region around X(t) has to be used to map a small region around Y(t). For this purpose, at least E+1 points of $M_{X}$ are needed [31,40], i.e., $\left[X\left(t_{1}\right),X\left(t_{2}\right),\ldots ,X\left(t_{E+1}\right)\right]$ ordered from the nearest to the farthest. The corresponding points $\left[Y\left(t_{1}\right),Y\left(t_{2}\right),\ldots ,Y\left(t_{E+1}\right)\right]$ are then used to estimate Y(t), as follows:

(4) $$\hat{Y}\left(t\right)|M_{X}=\sum_{i=1}^{E+1}w_{i}Y\left(t_{i}\right)$$

In the equation above, the weights $w_{i}$ are computed using the Euclidean distances between X(t) and the nearest E+1 points (‖·‖ indicates the Euclidean distance in $R^{E}$):

$$\begin{array}{cccc} w_{i} & =\frac{u_{i}}{\sum_{j=1}^{E+1}u_{j}}, & u_{i} & =\exp\left(-\frac{\|X\left(t\right)-X\left(t_{i}\right)\|}{\|X\left(t\right)-X\left(t_{1}\right)\|}\right) \end{array}$$

Finally, the time series reconstructed through the cross-mapping and the original series are compared through the Pearson correlation coefficient, in order to quantify the goodness of the estimate.

In this study, we applied the cross-mapping technique to compare the quality of PRV series extracted from two different PPG signals ($PPG_{f}$ and $PPG_{w}$), as surrogates of the HRV series are calculated from an ECG signal simultaneously acquired. For each session of the experimental protocol adopted in this study, we used the points of the attractor related to the PRV series ($PRV_{w}$ and $PRV_{f}$) to estimate the points of the attractor described by the HRV series obtained from the ECG signal ($HRV_{ECG}$). We computed the same process two times: the first time estimating $HRV_{ECG}$ using $PRV_{w}$, i.e., $HRV_{ECG|PPG_{w}}$, and the second time estimating $HRV_{ECG}$ using $PRV_{f}$, i.e., $HRV_{ECG|PPG_{f}}$. Each of the three attractors related to $HRV_{ECG}$, $PRV_{f}$, and $PRV_{w}$ were reconstructed after searching for the optimal time delay τ, as the first minimum of the mutual information function [41] (τ=5±1, median ± MAD). The three optimized values of τ were used to find the three optimized embedding dimensions *m*, using the False Nearest Neighbors (FNN) method [42] (m=7±3, median ± MAD). The maximum value among the three embedding dimensions *m* was chosen as embedding dimension *E* to compute the CM approach.

For each experimental session, two Pearson correlation coefficients, i.e., $\rho_{f}$ and $\rho_{w}$, were computed between the $HRV_{ECG}$ signal and the reconstructed $HRV_{ECG|PPG_{f}}$ and $HRV_{ECG|PPG_{w}}$, respectively. The higher the ρ coefficient value, the higher the quality of the $HRV_{PPG}$ signal, as a surrogate of $HRV_{ECG}$.

### 2.4. Statistical Analysis

The statistical analysis on the PPG and PRV signal quality indexes extracted using finger and wrist sensor location, was performed using the same non parametric statistical tests. The use of non-parametric tests was justified by the non-gaussian distribution of samples (p<0.05 from Shapiro–Wilk test).

Assuming that *x* is a generic index belonging to the vector of all the parameters described in Section 2.2 and Section 2.3, i.e., $x_{f}\in \left[kurt_{f},shannon_{f},r_{f},\rho_{f}\right]$ and $x_{w}\in \left[kurt_{w},shannon_{w},r_{w},\rho_{w}\right]$, we computed two different statistical analyses:

s1. A Friedman test was used to compare the $x_{f}$ and $x_{w}$ values considering four repeated measures for each subject (one for each experimental session). In post-hoc analysis, we compared the $x_{f}$ and $x_{w}$ of each singular experimental session, using a two-tailed Wilcoxon signed-rank test with false discovery rate (FDR) adjustment through the Benjamini–Yekuteli correction [43].
s2. A Friedman test with two repeated measures (i.e., $x_{f}$ and $x_{w}$) was applied to assess possible statistical differences among the *x* values of the four experimental sessions (n1, N, P, n2). As a post-hoc analysis, we performed a Wilcoxon test for each pair of experimental sessions (n1 vs. N, n1 vs. P, n1 vs. n2, N vs. P, N vs. n2, P vs. N2) considering both $x_{f}$ and $x_{w}$ singularly. In addition, in this case, FDR was controlled through the Benjamini–Yekuteli correction.

## 3. Results

### 3.1. Kurtosis and Shannon Entropy Results

**Figure 1:**
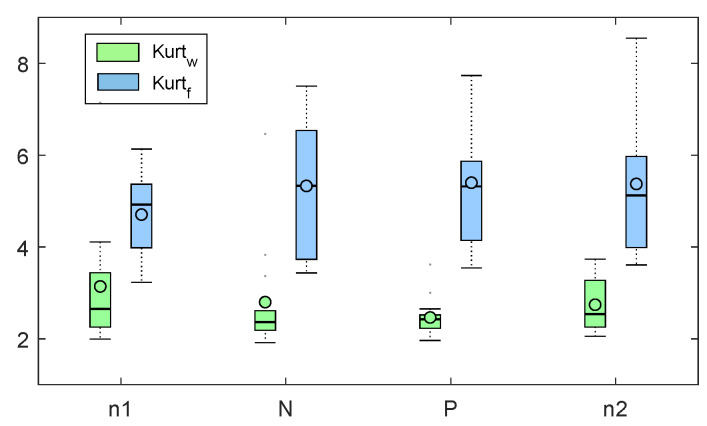
Boxplots related to $kurt_{f}$ (blue) and $kurt_{w}$ (green) values corresponding to the four experimental sessions (n1, N, P, n2).

As regards the PPG quality indexes, the wrist system showed to be corrupted by localized movement artifacts in 3 on 16 subjects. We decided to remove those subjects from the analysis since both the kurtosis and Shannon entropy resulted in outlier values. The median and median absolute values of kurtosis, across subjects, for the different sessions obtained by both finger and wrist system are shown in Figure 1. The s1 test as applied to kurtosis revealed a significant lower value in wrist system measures, with respect to finger system measurement (*p* < $1\times 10^{-12}$). This was confirmed across the four sessions by post hoc analysis. The result of s2 test as applied to kurtosis values, did not highlight any significant difference among the values obtained with the different recording sessions (*p* > 0.8). Figure reporting the boxplots related to test s2 are reported in Supplementary Materials (Figure S1).

**Figure 2:**
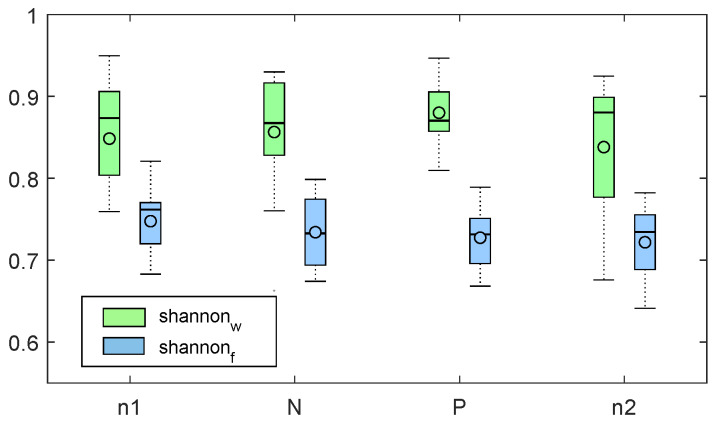
evBoxplots related to $shannon_{f}$ (blue) and $shannon_{w}$ (green) values corresponding to the four experimental sessions (n1, N, P, n2).

The median and median absolute values of Shannon entropy obtained with the two systems are reported in Figure 2. Shannon entropy was significantly higher in wrist system measurement in all the recording sessions (*p* < 5$\times 10^{-10}$). The recording sessions did not result in significantly different values (*p* > 0.9). Figure reporting the boxplots related to test s2 are reported in Supplementary Materials (Figure S2).

### 3.2. Statistical Correlation Results

Applying the statistical test s1 to the correlation coefficients $r_{f}$ and $r_{w}$, we did not find statistically significant differences. In all of the four sessions of the experiment, the median value of $r_{f}$ coefficients was higher with respect to $r_{w}$.

Concerning the results of statistical test s2, we did not find significant differences between $r_{f}$ and $r_{w}$ among the four experimental sessions (*p* > 0.05).

### 3.3. Cross-Mapping Results

**Figure 3:**
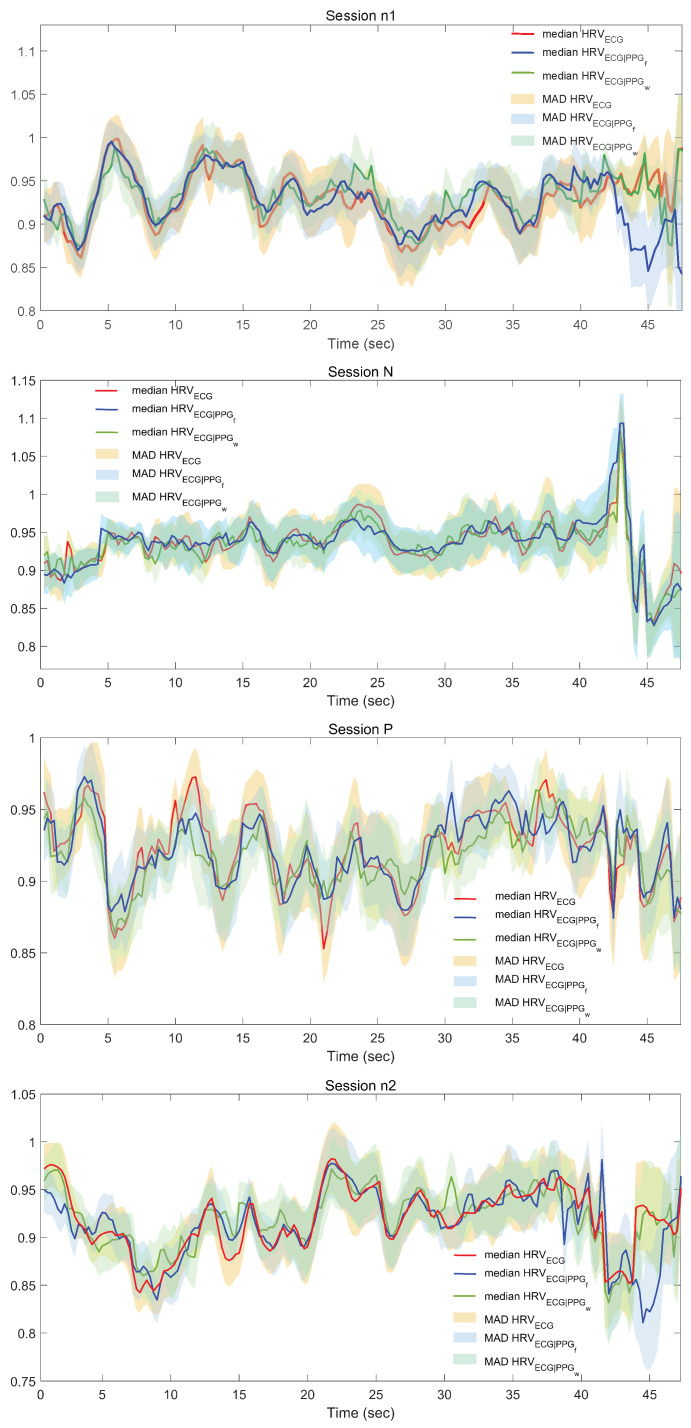
Trends of median and median absolute deviation (MAD) values of *HRV*${}_{ECG}$, *HRV*${}_{ECG|PPG_{f}}$, *HRV*${}_{ECG|PPG_{w}}$ as a function of the time for each experimental session (n1, N, P, n2). The time duration of the graphs is less than 60 s because by applying the cross-mapping (CM) method we are unable to reconstruct the last part of the signal (the points in the reconstructed phase space are n−(E−1)τ).

In Figure 3, we reported four graphs representative of the effects of applying the cross mapping method. We showed the trends of median and median absolute deviation (MAD) values of *HRV*${}_{ECG}$, *HRV*${}_{ECG|PPG_{f}}$, *HRV*${}_{ECG|PPG_{w}}$ as a function of the time for each experimental session (n1, N, P, n2).

**Figure 4:**
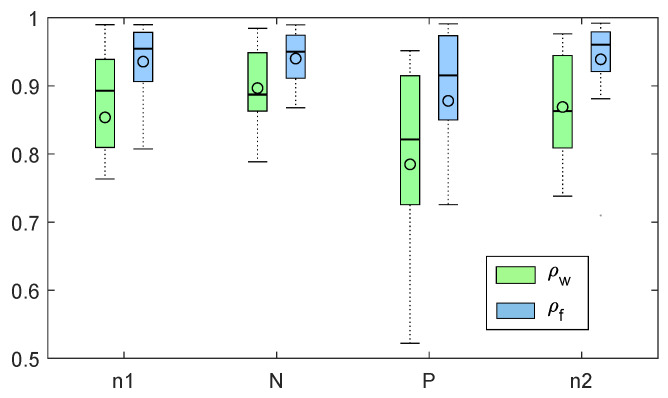
Boxplots related to $\rho_{f}$ (blue) and $\rho_{w}$ (green) values corresponding to the four experimental sessions (n1, N, P, n2).

After the application of the cross-mapping procedure, we calculated the values of the correlation coefficients between each $HRV_{ECG}$ referred to a single experimental session and the related $HRV_{ECG|PPG}$, i.e., $\rho_{w}$ and $\rho_{f}$, as described in Section 2.3.2. The median and median absolute values (MAD) of $\rho_{w}$ and $\rho_{f}$ obtained for each experimental session are reported in Figure 4. It is possible to note that higher correlation coefficient values are associated to the comparison between HRV extracted from ECG and finger-PPG signals with respect to the wrist-PPG signals.

After the computation of $\rho_{w}$ and $\rho_{f}$ values, we performed the two statistical analyses described in Section 2.4. The Friedman test of the s1 analysis confirmed that the $\rho_{f}$ index associated with finger sensors was significantly higher than $\rho_{w}$ values obtained considering the sensor on the wrist, with *p*-value = $9.27\times 10^{-8}$.

In addition, we found that such a trend was statistically significant in every single session, with *p*-values lower than 0.01 after Wilcoxon statistical tests. Concerning s2 analysis, we obtained a significant *p*-value = 0.023, which indicated a statistical difference in the correlation coefficient values among the four experimental sessions. As a post-hoc analysis, we practiced twelve Wilcoxon tests to compare each couple of sessions, six using $\rho_{f}$ values and six using $\rho_{w}$ values, as explained in Section 2.4. The results of post-hoc analysis, related to the *p*-values found with the Wilcoxon statistical tests corrected through FDR procedure, did not evidence statistically significant differences in the pairwise comparisons. Figure reporting the boxplots related to test s2 are reported in Supplementary Materials (Figure S3).

## 4. Discussion and Conclusions

The study of the reliability of PRV measurements has gained great interest in the last decade, as witnessed by numerous examples of PRV used as a HRV surrogate in various contexts. The cost-effectiveness and practicality of the PPG acquisition systems make it particularly convenient to use this methodology for monitoring healthy subjects as well as patients. Other crucial advantages of PPG devices are the easiness of connection with IoT technologies and their truly non-invasive approach.

Most of the previous studies that tested PRV accuracy and reported an acceptable agreement with HRV considered recordings under ideal conditions, such as from young subjects in resting state [21,44]. Moreover, promising findings were achieved in the diagnosis of obstructive sleep apnea, performing simultaneous ECG and PPG night-sleep recordings [45]. When tasks involving physical activity or mental stressors were studied, the disagreement between HRV and PRV analyses came up [21,46,47]. In such conditions, reflectance-mode PPG devices are less restrictive than transmission-mode PPG systems in terms of measurement sites in practice, but they are more prone to motion artifacts and signal quality can change more with sensor positioning [37,48]. At the same time, in reflectance-mode PPG the contact pressure can be very low, especially during exercise, and this cause a reduction of the measured signal amplitude. All these issues have to be considered also when PPG signals are acquired for long-term monitoring during the daily activities of the elderly and patients affected by cardiovascular diseases or with histories of cardiovascular disease. Discrepancies between HRV and PRV could be associated to movement artifacts, noise, but particularly to physiological processes, e.g., the stronger mechanical coupling between respiration and the thoracic vascular system during standing than supine position [49]. Another interesting physiological effect which can be noticed during both physical and mental tasks is the high arterial stiffness, that can reduce the similarity of PRV and HRV [50]. An increase of arterial stiffness was found also in stroke survivors when compared to controls during standing, and such condition was demonstrated to be accompanied by a low agreement between HRV and PRV [51]. Constant et al. considered a group of healthy children and found that the respiratory fluctuations in PPG-derived signals were more pronounced than in the ECG-based signal [24]. Due to this bias especially relevant in the high-frequency components, PRV was not recommended as a surrogate for HRV in studies comprising standing positions or participants with low HRV.

In this study, ECG and PPG signals were simultaneously acquired from healthy young subjects in sitting position, during a protocol of affective stimulation. The variability of HRV dynamics during this type of experimental protocols has been widely demonstrated in previous studies [3,52,53,54,55]. In the current study, participants were passively stimulated by showing pictures selected from the IAPS database [34]. The experimental protocol consisted of four one-minute long sessions. In the first and fourth sessions, neutral emotional pictures were displayed while in the second and third ones high-arousal images with negative and positive valence were shown, respectively. During each experimental session, the subjects were asked to watch ten images for 6 s each. From each subject, two PPG signals from the left index finger ($PPG_{f}$) and the left wrist ($PPG_{w}$) were recorded. The influence of sensor location on PPG and PRV signal quality was investigated in many previous studies in the literature, as reported in Section 1. According to the literature, we chose the finger site as gold-standard suggested for clinical PPG acquisitions, and the wrist as the most practical site that allows the use of smartwatches as monitoring devices [17,56,57,58]. The analysis of kurtosis revealed a lower value in the wrist system. In [19], this was considered as a sign of higher quality signal, since it might be related to a reduced impact of movement artifacts. On the other hand, Shannon entropy was found to be higher in the wrist. Such a result might be related to an increase of informative content of the signal acquired with this modality. It is important to underline the fact that Shannon entropy was estimated while keeping the number of bin fixed as in [19], and by adapting bin size in each recording to signal dynamics. With such an approach, possible outliers or movement artifact might cause the smaller, noise-free, PPG signal components to be described by a reduced number of bins, thus resulting in an overall decrease of Shannon entropy. The s2 test revealed no significant task-related effects on the observed quality indexes, i.e., PPG quality indexes were found indifferent across all of the recording sessions. In summary, we can state that the observed quality indexes show that the wrist provided better signal quality. Then, we proposed to compare the whole PRV dynamics in the phase space with HRV dynamics, using the cross-mapping approach [32]. Applying CM methodology, we were able to reconstruct HRV trajectories from PRV${}_{f}$ with more precision with respect to the results obtained by using PRV${}_{w}$. The correlation coefficients $\rho_{f}$ found after the comparison between the original $HRV_{ECG}$ and the reconstructed $HRV_{ECG|PPG_{f}}$ were significantly higher than the values of $\rho_{w}$ related to the comparison between the original $HRV_{ECG}$ and the reconstructed $HRV_{ECG|PPG_{w}}$ (see Figure 4). For both wrist and finger location, the median value of ρ coefficients decreased in the two arousing sessions, reaching the minimum value in the third experimental session, corresponding to the elicitation through positive images. The CM approach proposed allows obtaining a synthetic and parsimonious quality index of the PRV signal. We hypothesize that our proposed approach could represent a more unambiguous and robust approach for PRV quality assessment, with respect to the comparative evaluation of a plethora of features.

Our findings suggest that the study of nonlinear dynamics can unveil relevant differences among different devices or sensor locations. Moreover, the physiological changes caused by high-arousing emotional stimulation can lead to an increase in discrepancies between HRV and PRV, pointing out, especially in the case of the wrist, a remarkable disagreement compared to neutral emotional stimulation. In conclusion we can state that nonlinear dynamic approach highlighted that both PRVs from finger and wrist reported a good correlation coefficient with the HRV, i.e., greater than 0.8, even reaching values of 0.95, although finger, as easily expected, resulted to be more reliable. This remarkable agreement with HRV might justify the use of nonlinear methods for the analysis of PRV in univariate and multivariate manner [59].

## 5. Limitations and Future Work

Two limitations of our study are the age of the subjects (the participants were healthy and young) and their position while the signals were acquired (sitting still in front of a screen). These two conditions greatly reduce the possibility of movement artifacts and physiological disorders that can alter the signals. Future works will be addressed towards the application of existent PPG signal quality indexes and of cross-mapping approach to recordings acquired in groups of subjects of different age (including children and elderly), and during physical activity and protocols of intense affective stimulation (video, odor, or multimodal stimuli).

Furthermore, we will study the properties of the $HRV_{ECG|PPG}$ signals produced through the cross-mapping method, comparing them with the PRV signals. In this way, we will use our approach not only as an aid for assessing the quality of acquired PRV signals, but also as a new method of multivariate processing of cardiovascular signals. In fact, the signals derived from the application of cross-mapping technique by merging the information given by ECG and PPG signals, could contain new relevant insight in the study of cardiovascular health.

## Supplementary Materials

The following are available online at https://www.mdpi.com/1424-8220/20/11/3156/s1, Figure S1: Boxplots related to $kurt_{f}$ (blue) and $kurt_{w}$ (green) values in the whole experimental protocol. Figure S2: Boxplots related to $shannon_{f}$ (blue) and $shannon_{w}$ (green) values in the whole experimental protocol. Figure S3: Boxplots related to $\rho_{f}$ (blue) and $\rho_{w}$ (green) values in the whole experimental protocol.

## Abbreviations

The following abbreviations are used in this manuscript:

CM	Cross Mapping
HRV	Heart Rate Variability
ANS	Autonomic Nervous System
ECG	Electrocardiogram
PPG	Photoplethysmography
PRV	Pulse Rate Variability
IAPS	International Affective Picture System
MAD	Median Absolute Deviation
PSD	Power Spectral Density
