# Supplementary material for: Assessing the Quality of Heart Rate Variability Estimated from Wrist and Finger PPG: A Novel Approach Based on Cross-Mapping Method
Source: Sensors (Basel). 2020 Jun 2;20(11):3156. doi: 10.3390/s20113156 (PMC7309104; doi:10.3390/s20113156)
Supplement: Supplementary file 1 [file sensors-20-03156-s001.pdf]

sensors

MDPI

Article

Supplementary materials: Assessing the quality of Heart Rate Variability estimated from wrist and finger PPG: a novel approach based on cross-mapping method

Mimma Nardelli [1,2], Nicola Vanello [1,2], Guenda Galperti[1], Alberto Greco[1,2] and Enzo Pasquale Scilingo [1,2*]

[1] Bioengineering and Robotics Research Centre E. Piaggio, University of Pisa, Pisa 56122, Italy; m.nardelli@ing.unipi.it (M.N.); nicola.vanello@unipi.it (N.V.); alberto.greco@unipi.it (A.G.)
[2] Dipartimento di Ingegneria dell'Informazione, University of Pisa, Pisa 56122, Italy; guendagalperti@gmail.com
* Correspondence: enzo.scilingo@unipi.it (E.P.S.)

Received: 11 April 2020; Accepted: 31 May 2020; Published: date

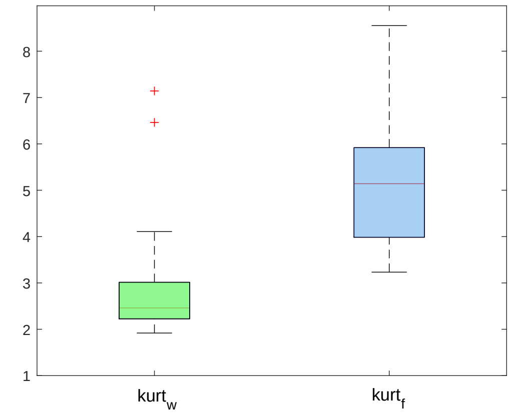

Figure S1. Boxplots related to $kurt_f$ (blue) and $kurt_w$ (green) values in the whole experimental protocol.

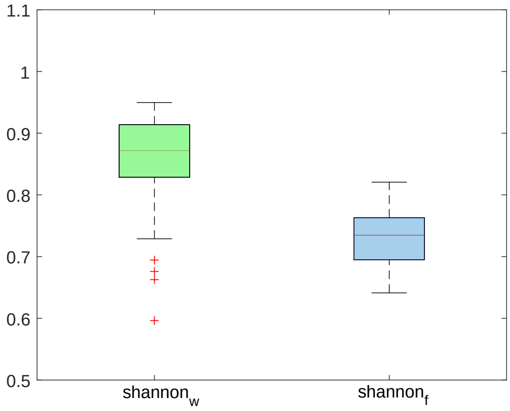

Figure S2. Boxplots related to $shannon_f$ (blue) and $shannon_w$ (green) values in the whole experimental protocol.

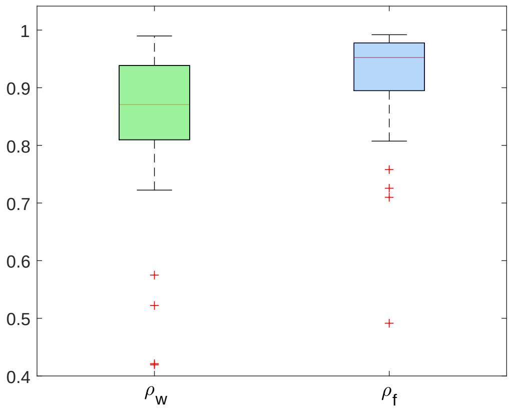

Figure S3. Boxplots related to ρ_f (blue) and ρ_w (green) values in the whole experimental protocol.

© 2020 by the authors. Licensee MDPI, Basel, Switzerland. This article is an open access article distributed under the terms and conditions of the Creative Commons Attribution (CC BY) license (http://creativecommons.org/licenses/by/4.0/).
